# The role of oxytocin and alexithymia in the therapeutic process

**DOI:** 10.3389/fpsyg.2014.01074

**Published:** 2014-09-23

**Authors:** Markus Quirin, C. Sue Carter, Regina C. Bode, Rainer Düsing, Elise L. Radtke, Mattie Tops

**Affiliations:** ^1^Department of Personality Research, Institute of Psychology, Osnabrück UniversityOsnabrück, Germany; ^2^Behavioral Neuroscience at the Research Triangle Institute International, University of North CarolinaChapel Hill, NC, USA; ^3^Department of Clinical Psychology, Faculty of Psychology and Education, VU University AmsterdamAmsterdam, Netherlands

**Keywords:** oxytocin, memory reconsolidation, therapeutic alliance, stress, emotions

Based on an extant literature review Lane et al. propose that therapeutic success results from changing conflictual, often non-conscious, emotional memories. Specifically, existent internal autobiographical models based on negative emotional memories become activated within the context of a positively colored therapeutic dyad and are reconsolidated as new, more positively colored internal models. Based on empirical evidence, we would like to extend this model by describing how the reconsolidation process may be facilitated on at least three levels of neurobiological functioning. Those three levels relate to an overarching principle in the function of the neuropeptide oxytocin to facilitate the construction and selection of positive internal models of social relationships and support.

First, oxytocin facilitates social bonds and trust as well as counteracts stress by dampening activity of physiological stress-systems and increasing parasympathic activity, and regulates glucocorticoid receptor expression in the hippocampus (Carter, 1998, see also Quirin et al., 2011). As such, oxytocin might facilitate the establishment of a positive therapeutic alliance and a relaxed atmosphere during therapy, which makes it easier for patients to awaken conflictual memories and tolerate concomitantly resurfacing negative emotions.

Second, oxytocin is implicated in the ontogenetic development of the neocortex and thus plays an important role in the construction of internal models (Carter, 2014). For example, it stimulates genetic regulation of the growth of the neocortex and the maintenance of the blood supply to the cortex—conditions that are prerequisites for the formation and sustentation of internal autobiographical models. In humans, oxytocin also facilitates cognitive control that helps individuals to bring behavior in line with internal models (Tops et al., 2013a). This is important since behavior control is a highly valued aspect of therapeutic success, which remained unaddressed by Lane and colleagues.

Third, oxytocin unfolds effects on a higher level of brain functioning. Specifically, while increasing familiarity and trust within a social context, oxytocin promotes the assimilation of novel emotional experiences into internal models (e.g., Tops et al., 2013b; for a review, see Tops et al., 2013a). This process takes place on the basis of semantic elaboration and verbalization of emotions as supported by parts of the left inferior frontal gyrus (Tops et al., 2014). This is compatible with the model by Lane et al., according to which the ability to symbolize and explain traumatic emotional memories in words helps individuals assimilate these memories into their narrative internal models of themselves and the world.

Based on these findings, oxytocinergic interventions may be developed to unfold their potential to facilitate therapeutic change (Harris and Carter, 2013). Such interventions may be especially helpful for so-called alexithymic patients, that is, individuals suffering difficulties in becoming aware of and in verbalizing their emotions (Taylor and Bagby, 2004). In fact, initial evidence indicates that intranasal oxytocin application increases verbal emotional sharing (Lane et al., 2013) and, especially in alexithymic patients, emotion recognition (Luminet et al., 2011). This is of high importance since alexithymia is increased in a broad array of mental disorders such as depression, anxiety, posttraumatic stress disorder, schizophrenia and autism spectrum disorder, and predicts poor therapy outcome (Samur et al., 2013). Moreover, in autism spectrum disorder patients, deficiencies that were found to be reduced after intranasal oxytocin application, such as decreased eye-fixation, relate to alexithymia scores but not to autism symptoms, suggesting that it may be alexithymic deficiencies that are reduced by oxytocin (Bird et al., 2011).

Characteristics such as alexithymia may not only relate to larger oxytocin-induced improvements in emotion recognition and perception skills, but also to aversive responses to those improvements because they hinder habitual defenses in those individuals. Results suggest that affiliative effects of oxytocin might be limited to situations in which the subject already feels a sense of social inclusion (De Dreu, 2012) or more generally, when interaction partners are familiar or when there is opportunity to familiarize oneself with interaction partners (Tops et al., 2013a,b, 2014). Oxytocin facilitates the perception of positive and supportive social cues (Theodoridou et al., 2009; Guastella and MacLeod, 2012) allowing for positive familiarization and habituation of social uneasiness (“familiarization-habituation response;” Tops et al., 2013b). Alexithymia, as well as fearful attachment and self-criticism, are associated with negative responses to and avoidance of positive social cues (Dewitte and De Houwer, 2008; Gilbert et al., 2012). In a study of borderline personality disorder patients and healthy controls, fearful attachment mediated decreased trust and cooperation after intranasal oxytocin (Bartz et al., 2011a). Similarly, non-clinical participants higher in self-criticism, and those lower in attachment security, had less positive experiences of compassion-focused imagery after intranasal oxytocin than placebo (Rockliff et al., 2011)^1^. This suggests that facilitation of the perception of positive and supportive cues by oxytocin may be aversive for individuals who defensively prefer to avoid such cues to prevent painful experiences of loosing love and support (Gilbert et al., 2012). Potential aversive responses to oxytocin treatment means that further research is needed regarding the suitability and optimal strategies of oxytocin treatment to facilitate therapy in specific target groups.

In summary, oxytocin might act as a facilitator or even mediator for the therapeutic effects described by the authors of the target article: Since oxytocin facilitates both the establishment of positive, trustful relationships and the assimilation of emotional information into internal models, it may constitute a central neurobiological promoter of the reconsolidation of adverse or even traumatic memories within the context of psychotherapy. However, personality traits, disorder severity, context, and potentially other factors may moderate effects of oxytocin on psychosocial functioning (Bartz et al., 2011b) and have to be taken into account when thinking about how to implement oxytocinergic interventions. Moreover, individuals who normally avoid or react aversively to positive and supportive social cues need special attention in the context of therapy and oxytocin augmentation.

## Conflict of interest statement

The authors declare that the research was conducted in the absence of any commercial or financial relationships that could be construed as a potential conflict of interest.

## Footnotes

^1^In this double-blind randomized within-subject study all subjects took part in an identical placebo and oxytocin treatment session. Interestingly, if the samples were split according to high and low self-criticism score, effects were only present for high self-critics who showed significantly more resistance to compassion-focused imagery when given oxytocin first. This suggests that intranasal oxytocin may have amplified perception of positive and supportive social cues in the novel social context of the first session, reflecting amplification of oxytocin's natural function in familiarization-habituation responses (Tops et al., 2013b). In turn, perception of supportive positive social cues increases resistance in high self-critical individuals.
